# Epidemiology and risk factors of infective endocarditis in a tertiary hospital in China from 2007 to 2016

**DOI:** 10.1186/s12879-020-05153-w

**Published:** 2020-06-18

**Authors:** Zhenzhu Wu, Yi Chen, Tingting Xiao, Tianshui Niu, Qingyi Shi, Yonghong Xiao

**Affiliations:** 1 State Key Laboratory for Diagnosis and Treatment of Infectious Diseases, National Clinical Research Center for Infectious Diseases,Collaborative Innovation Center for Diagnosis and Treatment of Infectious Diseases, the First Affiliated Hospital, College of Medicine, Zhejiang University, 79 Qingchun Road, Hangzhou, Zhejiang Province China; 2 The Second Affiliated Hospital and Yuying Children’ Hospital of Wenzhou Medical University, Wenzhou, China

**Keywords:** Infective endocarditis, Epidemiology, Risk factors, Mortality

## Abstract

**Background:**

To explore the trends in epidemiology and the risk factors related to the prognosis of infective endocarditis in a tertiary hospital over the past ten years.

**Methods:**

A retrospective cohort study was performed. A total of 407 consecutive patients who were admitted with infective endocarditis were included. The clinical characteristics and the risk factors related to the prognosis of infective endocarditis during this period were analyzed.

**Results:**

A total of 407 patients with infective endocarditis were included, the average age was 48 ± 16 years old with an increasing trend and in-hospital mortality rate was 10.6% and one-year mortality rate was 11.3%. Among patients with underlying heart disease, congenital heart disease was the most common (25.8%), followed by rheumatic heart disease (17.0%) which showed a decreased trend during this period (P < 0.001). There were 222(54.5%) patients with positive blood cultures results and *Streptococci* (24.6%) was the main pathogens with an increasing trend. There were 403 patients (99%) with surgical indications, but only 235 patients (57.7%) received surgical treatment. Hemodialysis (P = 0.041, OR = 4.697, 95% CI 1.068–20.665), pulmonary hypertension (P = 0.001, OR = 5.308, 95% CI 2.034–13.852), Pitt score ≥ 4 (*P* < 0.001, OR = 28.594, 95% CI 5.561–148.173) and vegetation length>30 mm (P = 0.011, OR = 13.754, 95% CI 1.832–103.250) were independent risk factors for in-hospital mortality.

**Conclusions:**

There were no significant changes in the overall incidence of infective endocarditis, but the clinical features of infective endocarditis had slightly changed during the past ten years. *Streptococci* infective endocarditis was still the predominant. Patients with hemodialysis, pulmonary hypertension, Pitt score ≥ 4 and vegetation length>30 mm had an worse in-hospital outcome.

## Background

Infective endocarditis (IE) is an infectious disease involving the heart valve or endocardium caused by causative microorganisms. It is a severe disease with high morbidity and mortality [1]. Serious complications such as heart failure and cerebral embolism are common. In developed countries, the annual incidence is between 3 and 9 patients per 100,000 persons with a slight increase between 1970 and 2013 [1, 2]. Although the improvement of prevention, the progress of antibiotic therapy, the development of imaging technology and the intervention with surgery, the mortality is still up to 15–30% [3]. Over the past two decades, with the increase in life expectancy, the increased use of cardiac implant devices, and the frequency in invasive procedures, the epidemiology of IE in developed countries such as the Europe and the United States has changed significantly: older patients with IE gradually increased, prosthetic valve endocarditis (PVE) and cardiac device–related endocarditis increased, and *Staphylococci* has become the most predominated pathogen [4].

However, studies from developing countries such as Turkey showed a different epidemiological character [5]. In their study, although *Staphylococcal* IE was increasing, *Streptococci* was still the most predominant pathogen; rheumatic heart disease (RHD) and congenital heart disease (CHD) were still the most common underlying heart diseases, and the number of patients undergoing surgical treatment was increasing [5, 6]. In this study, we analyzed the changes in clinical features of IE over the past 10 years in a tertiary teaching hospital to explore the epidemiological trend and the risk factors related to the prognosis of IE.

## Methods

### Patients

A retrospective, consecutive case-series analysis was organized and performed in the First Affiliated Hospital of Zhejiang University, Hangzhou, China. Patients with a clinical diagnosis of IE from January 1, 2007 to December 31, 2016 were reviewed. The Modified Duke criteria [7] was used to evaluate patients reviewed. Patients who were admitted to the hospital more than one time for the same pathogen during the study period were considered as one case.

### Clinical parameters and study design

The data were retrieved from the Electronic Medical Record. The data included in the analysis were patient demographic information, underlying heart diseases, comorbidities, clinical manifestations, complications, causative microorganisms, echocardiographic demonstrations, treatments and outcomes. The following data analysis strategies were conducted: (1) The changing trend of clinical characteristics in IE was analyzed with every two-year time period; (2) To explore the prognostic risk factors, a comparison between the survival group and the death group was conducted.

### Definition

Infective endocarditis was categorized based on disease types according to the 2015 European Society of Cardiology (ESC) guidelines for the management of infective endocarditis as native-valve endocarditis, prosthetic-valve endocarditis, and cardiac device–related endocarditis [7].

Embolic events were diagnosed by imaging techniques including cerebral embolism and non-central nervous system embolism.

Heart failure was diagnosed according to the Framing-ham criteria [8]: major criteria were physician assessment of neck-vein distension, rales, S3 gallop, venous pressure > 16 cm of water, hepatojugular reflux, and weight loss of 4.5 kg in 5 days due to diuretic therapy, and minor criteria were ankle edema, night cough, dyspnea on exertion, hepatomegaly, tachycardia and weight loss. “Definite Heart failure” was defined as having at least two major criteria, or one major criterion and two minor criteria.

Renal insufficiency was defined as endogenous creatinine clearance rate (Ccr) < 60 mL/min/1.73 m2.

Pulmonary hypertension was defined by a resting mean pulmonary artery pressure ≥ 25 mmHg, pulmonary artery wedge pressure < 15 mmHg, and pulmonary vascular resistance > 3 Wood Units by echocardiography.

Surgical indications were based on the 2015 ESC guidelines for the management of infective endocarditis [7] including: heart failure, uncontrolled infection, vegetation > 10 mm or evidence of vegetation embolic event.

Previous antibiotic use referred to the use of antibiotics for > 72 h at any point 2 weeks prior to IE diagnosis.

Transthoracic echocardiogram (TTE) was performed routinely in all patients. Transesophageal echocardiogram (TEE) was used to detect cases with negative TTE results. Blood culture was performed in all the patients with aerobic, anaerobic and fungal blood cultures, but blood culture for the HACEK group (*Haemophilus spp, Aggregatibacter spp, Cardiobaterium hominis, Eikenella corrodens,* and *Kingella kingae*) and *anti-legionella*, *mycoplasma* and *bartonella* anti-body tests as well as PCR test were not performed.

The main outcome was in-hospital all-cause mortality and one-year all-cause mortality. The one-year follow-up data were collected from the patients’ latest visits to the hospital.

### Statistical analysis

The trends in clinical characteristics was analyzed using the log-linear Poisson regression model. Univariate analysis was performed using the Pearson’s χ^2^ test or Fisher’s exact tests as appropriate for categorical variables and the independent Student’s t-test or the Rank sum test as appropriate for continuous variables. Categorical variables were expressed as frequencies and percentages of the specified group and continuous variables were reported as averages with standard deviations or medians and interquartile ranges. After univariate analysis, variables with *P* < 0.05 were included in multivariate analysis to identify predictors for in-hospital mortality among IE patients. Odds ratios (OR) with 95% confidence interval (CI) were calculated in logistic regression. All tests were 2-tailed, and *P* < 0.05 was considered statistically significant. All analyses were performed using SPSS version 23 statistical software.

## Results

### Patient enrollment

In the study, there were 409 patients with IE and 2 patients whose blood culture results were unreachable were excluded. A total of 407 IE patients were included. There were 378 (92.8%) patients taking part in the study completed the one-year follow up after infective endocarditis diagnosis. There were 43 patients (10.6%) died during hospitalization and 46 patients (11.3%) died in the one-year follow-up.

### The clinical characteristics of patient with IE

**Fig. 1:**
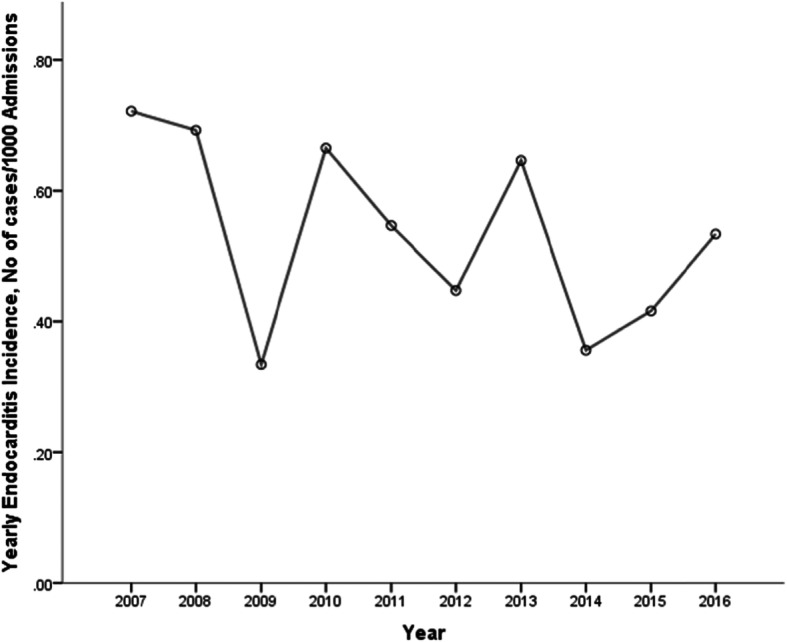
Incidence of infective endocarditis from 2007 to 2016 in hospitalized patients. The annual incidence of IE were between 0.33 and 0.72 patients per 1000 admissions and the incidence was stable over this period (Linear regression showed *P* = 0.168)

Among the 407 patients, 262 patients were male (64.4%).The average age was 48 ± 16 years old with an increasing trend during this period (*P* = 0.001) **(**Table 1**).** The annual incidence of IE were between 0.33 and 0.72 patients per 1000 admissions and the incidence was stable over this period **(**Fig. 1**)**.

**Table 1:** The clinical characteristics of infective endocarditis and changing trends by years from 2007 to 2016

Variable, n(%)	Total *n* = 407	2007–2008 *n* = 62	2009–2010 *n* = 63	2011–2012 *n* = 78	2013–2014 *n* = 89	2015–2016 *n* = 115	*P* value
Male	262 (64.4)	40 (64.5)	42 (66.7)	53 (67.9)	56 (62.9)	71 (61.7)	.513
Age,mean ± SD	48 ± 16	45 ± 15	42 ± 15	47 ± 16	50 ± 16	52 ± 16	.001
**Comorbidities**							
Hypertension	82 (20.1)	9 (14.5)	9 (14.3)	11 (14.1)	20 (22.5)	33 (28.7)	.005
Diabetes mellitus	37 (9.1)	3 (4.8)	5 (7.9)	6 (7.7)	8 (9.0)	15 (13.0)	.078
Hepatitis B virus infection	18 (4.4)	1 (1.6)	2 (3.2)	5 (6.4)	6 (6.7)	4 (3.5)	.501
Chronic organ disease	23 (5.7)	3 (4.8)	3 (4.8)	2 (2.6)	6 (6.7)	9 (7.8)	.260
Cancer	12 (2.9)	2 (3.2)	2 (3.2)	4 (5.1)	2 (2.2)	2 (1.7)	.474
No-underlying heart disease	186 (45.7)	18 (29.0)	29 (46.0)	42 (53.8)	40 (44.9)	57 (49.6)	.044
Underlying heart disease	221 (54.3)	44 (71.0)	34 (54.0)	36 (46.2)	49 (55.1)	58 (50.4)	.044
Congenital heart disease	105 (25.8)	17 (27.4)	16 (25.4)	18 (23.1)	26 (29.2)	28 (24.3)	.870
Rheumatic heart disease	69 (17.0)	22 (35.5)	14 (22.2)	12 (15.4)	6 (6.7)	15 (13.0)	<.001
Previous cardiac surgery	44 (10.8)	11 (17.7)	6 (9.5)	6 (7.7)	15 (16.9)	6 (5.2)	.086
Degenerative heart disease	13 (3.2)	0	0	2 (2.6)	2 (2.2)	9 (7.8)	.001
Hospital stay, mean ± SD	25 ± 22	31 ± 31	32 ± 25	23 ± 17	23 ± 24	21 ± 15	.001
Duration of Symptoms before echocardiography median (IQR),days	13 (4–35)	17 (7–48)	14 (6–30)	15 (4–40)	9 (3–50)	15 (4–31)	.542
Duration of symptoms before diagnosis, median (IQR),days	25 (12–62)	26 (12–68)	24 (9–42)	25 (14–62)	28 (10–75)	26 (13–62)	.434
Previous antibiotic use	321 (81.7)	48 (81.4)	58 (92.1)	65 (90.3)	76 (85.4)	74 (67.3)	.001
Intravenous drug abuse	3 (0.7)	1 (1.6)	1 (1.6)	0	0	1 (0.9)	.556
**Symptoms and signs**							
Fever	365 (89.7)	57 (91.9)	57 (90.5)	70 (89.7)	83 (93.3)	98 (85.2)	.233
Anemia	222 (54.5)	38 (61.3)	43 (68.3)	43 (55.1)	58 (65.2)	40 (38.4)	<.001
Osler nodule	7 (1.7)	2 (3.2)	0	3 (3.8)	2 (2.2)	0	.288
Janeway lesions or nailbed bleeding	4 (0.9)	1 (1.6)	3 (4.8)	0	0	0	.060
TEE	52 (12.8)	3 (4.8)	4 (6.3)	5 (6.4)	8 (9.0)	32 (27.8)	<.001
Infection site							.342
Left-side	356 (87.5)	53 (85.5)	54 (85.7)	67 (85.9)	79 (88.8)	103 (89.6)	
Right-side	31 (7.6)	7 (11.3)	3 (4.8)	8 (10.3)	4 (4.5)	9 (7.8)	
Left+ Right-side	20 (4.9)	2 (3.2)	6 (9.5)	3 (3.8)	6 (6.7)	3 (2.6)	
Infection valve							>.05
Single valve	295 (72.5)	38 (61.3)	46 (73.0)	55 (70.5)	58 (65.2)	98 (85.2)	.002
Mitral valve	134 (32.9)	16 (25.8)	20 (31.7)	24 (30.8)	30 (33.7)	44 (38.3)	
Aortic valve	143 (35.1)	18 (29.0)	23 (36.5)	25 (32.1)	27 (30.3)	50 (43.5)	
Multi-valve, n(%)	79 (19.4)	13 (21.0)	15 (23.8)	13 (16.7)	24 (27.0)	14 (12.2)	
Endocardial or arterial intima	33 (8.1)	11 (17.7)	2 (3.2)	10 (12.8)	7 (7.9)	3 (2.6)	
**Type of IE**							
Native valve IE	370 (90.9)	50 (80.6)	58 (92.1)	72 (92.3)	77 (86.5)	113 (98.3)	.002
Prosthetic valve IE or cardiac device–related IE	37 (9.1)	12 (19.3)	5 (7.9)	6 (7.7)	12 (13.5)	2 (1.7)	.002
**Complications**							
Embolic events	125 (30.7)	14 (22.6)	16 (25.4)	16 (20.5)	34 (38.2)	45 (39.1)	.003
Cerebral embolism	74 (18.2)	6 (9.7)	7 (11.1)	12 (15.4)	18 (20.2)	31 (27.0)	.001
Heart failure	215 (52.8)	31 (50.0)	31 (49.2)	43 (55.1)	51 (57.3)	59 (51.3)	.661
Vegetation	350 (86.0)	44 (71.0)	54 (85.7)	62 (79.5)	82 (92.1)	108 (93.9)	<.001
Vegetation length							<.001
≤10 mm	90 (22.1)	9 (14.5)	12 (19.0)	16 (20.5)	21 (23.6)	32 (27.8)	
>10 mm<20 mm	197 (48.4)	25 (40.3)	31 (49.2)	31 (39.7)	52 (58.4)	58 (50.4)	
≥20 mm ≤ 30 mm	51 (12.5)	8 (12.9)	10 (15.9)	14 (17.9)	6 (6.7)	13 (11.3)	
>30 mm	12 (2.9)	2 (3.2)	1 (1.6)	1 (1.3)	3 (3.4)	5 (4.3)	
Valvular perforation	72 (17.8)	3 (4.9)	7 (11.1)	11 (14.3)	21 (23.6)	30 (26.1)	<.001
Perivalvular abscess	30 (7.4)	0	1 (1.6)	3 (3.9)	12 (13.5)	14 (12.2)	<.001
Positive rate blood culture	222 (54.5)	41 (66.1)	33 (52.4)	36 (46.2)	44 (49.4)	68 (59.1)	.663
Streptococci	100 (24.6)	17 (27.4)	9 (14.3)	15 (19.2)	20 (22.5)	39 (33.9)	.028*
Staphylococci	80 (19.7)	14 (22.6)	17 (27.0)	12 (15.4)	15 (16.9)	22 (19.1)	.668*
Enterococci	5 (1.2)	1 (1.6)	1 (1.6)	1 (1.3)	1 (1.1)	1 (0.9)	.051*
Surgical indications	403 (99.0)	60 (96.8)	63 (100.0)	76 (97.4)	89 (100.0)	115 (100.0)	.061
Surgical treatment	235 (57.7)	36 (58.1)	34 (54.0)	44 (56.4)	58 (65.2)	63 (54.8)	.900
In-hospital mortality	43 (10.6)	12 (19.4)	3 (4.8)	2 (2.6)	10 (11.2)	16 (13.9)	.910
One-year mortality	46 (11.3)	13 (21.0)	3 (4.8)	4 (5.1)	10 (11.2.)	16 (13.9)	.868

* percentage accouting for the positive results in each group

*SD* Standard deviation, *IQR* Interquartile range, *IE* Infective endocarditis, *TEE* Transesophageal echocardiography

There were 186 patients (45.7%) without underlying heart disease and the proportion increased over this time (*P* = 0.044). CHD was the most common underlying heart disease, accounting for 25.8% of all patients, and the proportion of RHD decreased yearly during this period (*P* < 0.001). Patients with degenerative valvular disease (DVD) accounted for 3.2% with a growing trend (*P* = 0.001). Native valve IE was still the most dominant, accounting for 90.9% and the proportion increased over this time (*P* = 0.002). There were 37 patients (9.1%) with diabetes mellitus and 18 patients (4.4%) with hepatitis B virus infection. Except for hypertension with a significant increase in the proportion (*P* = 0.005), the proportion of other comorbidities did not change significantly. There were 215 patients (52.8%) with heart failure and 74 patients (18.2%) with cerebral embolism. The proportion of cerebral embolism was significantly increased during this period (*P* = 0.001).

TEE was performed on 52 patients (12.8%) and 30 patients (7.4%) were diagnosed after TEE. The detection rate of vegetation was 86.0% with a significant growth trend (*P* < 0.001). There were 72 patients (17.8%) with valve perforation and 30 patients (7.4%) with perivalvular abscess. Left-side IE was the most common with 356 patients (87.5%). A single valve being affected was more common, with 295 patients (72.5%), among which the mitral valve was affected in 134 patients (32.9%) and the aortic valve was affected in 143 patients (35.1%).

Blood cultures were performed in all patients with IE. There were 222 (54.5%) positive blood cultures results. Patients with negative blood culture were diagnosed as definite IE by the following criteria: 173 patients had positive echocardiographic findings plus three minor criterias, and 12 patients were identified by positive histopathological findings. *Streptococci* was the main pathogen, accounting for 24.6% with an increasing trend, followed by *Staphylococci*, accounting for 19.7%. There were 40 patients (9.8%) of *Staphylococcus aureus* IE, including 26 patients (6.4%) of *Methicillin-resistant Staphylococcus aureus* (MRSA) IE. Other pathogens identified by blood cultures were: Gram-negative bacilli in 28, *Enterococci* in 5, *Corynebacterium* in 3 and *Kocuria roseus* in 1. Polymicrobial infection was identified in 5 patients.

There were 403 patients (99.0%) with surgical indications but only 235 patients (57.7%) with surgical treatment. Among those with surgical indications, 215 patients (52.8%) with heart failure, 125 patients (30.7%) with embolic events, 260 patients (63.9%) with vegetation > 10 mm, and 43 patients (10.6%) with uncontrolled infection. There were 68 patients (16.7%) with one surgical indication and 335 patients (82.7%) with more than one surgical indications.

### The risk factors for in-hospital mortality

Univariate analysis found that age, previous heart valve surgery, hemodialysis, hypertension, heart failure, cerebral embolism, cerebral hemorrhage, arrhythmia, hepatic insufficiency, renal insufficiency, pulmonary hypertension, vegetation length>30 mm, prosthetic valve or pacemaker valve IE, *Staphylococcus aureus* infection, Pitt score ≥ 4,surgical treatment were related to the in-hospital mortality **(**Table 2**)**. Multifactor analysis found that hemodialysis (*P* = 0.041, OR = 4.697, 95% CI 1.068–20.665), pulmonary hypertension (*P* = 0.001, OR = 5.308, 95% CI 2.034–13.852), Pitt score ≥ 4 (*P* < 0.001, OR = 28.594, 95% CI 5.561–148.173) and vegetation length>30 mm (*P* = 0.011, OR = 13.754, 95% CI 1.832–103.250) were independent risk factors for in-hospital mortality **(**Table 3**).**

**Table 2:** Risk factors of in-hospital outcome in patients with infective endocarditis (univariate analysis)

Variable, n (%)	In-hospital outcome		*P* value
	Survival *n* = 364	Death *n* = 43	
Age,mean ± SD	48 ± 16	53 ± 16	.047
Man	237 (65.1)	25 (58.1)	.367
Underlying heart disease	194 (53.3)	27 (62.8)	.237
Rheumatic heart disease	62 (17.0)	7 (16.3)	.901
Congenital heart disease	95 (26.1)	10 (23.3)	.687
Previous cardiac surgery	33 (9.1)	11 (25.6)	.001
Degenerative heart disease	13 (3.6)	0	.377
Others	18 (4.9)	4 (9.3)	.273
**Comorbidities**			
Chronic obstructive pulmonary disease	5 (1.4)	1 (2.3)	.491
Cancer	11 (3.0)	1 (2.3)	>.05
Hemodialysis	9 (2.5)	5 (11.6)	.002
Hepatitis B virus infection	16 (4.4)	2 (4.7)	>.05
Liver cirrhosis	4 (1.1)	0	>.05
Hypertension	68 (18.7)	14 (32.6)	.032
Diabetes mellitus	32 (8.8)	5 (11.6)	.541
**Complications**			
Heart failure	179 (49.2)	36 (83.7)	<.001
Intracranial infection	15 (4.1)	2 (4.7)	.698
Cerebral embolism	55 (15.1)	19 (44.2)	<.001
Cerebral hemorrhage	16 (4.4)	7 (16.3)	.001
Arrhythmia	65 (17.9)	15 (34.9)	.008
Hepatic insufficiency	57 (15.7)	15 (34.9)	.002
Renal insufficiency	60 (16.5)	21 (48.8)	<.001
Pulmonary hypertension	92 (25.6)	24 (57.1)	<.001
Valvular perforation	64 (17.7)	8 (18.6)	.881
Perivalvular abscess	26 (7.2)	4 (9.3)	.544
Vegetation length			<.001
≤10 mm	87 (23.9)	3 (7.0)	
>10 mm<20 mm	177 (48.6)	20 (46.5)	
≥20 mm ≤ 30 mm	46 (12.6)	5 (11.6)	
>30 mm	4 (1.1)	8 (18.6)	<.001
Vegetation extent	142 (39.0)	16 (37.2)	.819
**IE type**			.004
Native valve IE	337 (92.6)	33 (76.7)	
Prosthetic valve IE or cardiac device–related IE	27 (7.4)	10 (23.3)	.001
Previous antibiotic use	291 (82.4)	30 (75.0)	.249
**Causative organism**			.049
Streptococci	95 (26.1)	5 (11.6)	
Staphylococci	69 (19.0)	11 (25.6)	
*Staphylococcus aureus*	32 (8.8)	8 (18.6)	<.001
Pitt score			<.001
<4	359 (98.6)	23 (53.5)	
≥4	5 (1.4)	20 (46.5)	
Surgical treatment	221 (60.7)	14 (32.6)	<.001

*SD* Standard deviation, *IE* Infective endocarditis

**Table 3:** Risk factors of in-hospital outcome in patients with infective endocarditis (multivariate analysis)

Variable	*P* value	OR	95%CI
Male	.413		
Cerebral embolism	.137		
Hemodialysis	.041	4.697	1.068–20.665
Pulmonary hypertension	.001	5.308	2.034–13.852
Causative organism	.544		
Staphylococcus aureus	.422		
Pitt score ≥ 4	<.001	28.594	5.561–148.173
Vegetation length>30 mm	.011	13.754	1.832–103.250
Prosthetic valve IE or cardiac device–related IE	.064		
Surgical treatment	.325		

*OR* odds ratios, *CI* confidence interval

## Discussion

Over the past decades, the epidemiology of IE has changed due to changes in demographic characteristics and risk factors. Researches from developed countries have showed that the incidence of IE has increased in the past decades [4]. Cresti et al. [9] found the incidence was 4.6/100,000 person-years with a significant linear increase between 1998 and 2014. Keller et al. [10] found that the incidence of IE in Germany was 11.6/100,000 person-years between 2005 and 2015, and the incidence increased continuously during the study period, especially in the last five years. However, in our study, we found that the incidence of IE was between 0.33 and 0.72 patients per 1000 admissions and the incidence remained stable during the period, which was lower in comparison with the developed countries (approximately 1–1.3 patients per 1000 hospital admissions) [4, 9] .The difference in incidence of IE from different studies may be related to the geographical location of the study, the time period selected, and the difference in diagnostic techniques of different institutions.

In our study, we found that the mean age at the time of IE episodes were younger than those reported from developed countries, although the mean age increased during this period. The proportion of old patients with IE increased gradually in this period, which was more obvious in developed countries [9, 11]. Erichsen et al. [12] found that the incidence increased substantially for elderly IE patients between 1994 and 2011, with the highest incidence rate of 3.38 for patients more than 80 years old at IE onset. In Oliver et al.’ s report [13], 49% of IE patients were over 65 years old and 11.2% were over 80 years old. The changes in the age of IE onset may owing to the aging population. And as a result of the older onset age, patients with IE in the latter part of the study period were more likely to have comorbidities compared with patients in the earlier part of the study period.

We found that CHD had become the most common underlying heart disease for patients with IE in our study. This result was consistent with other researches from China, where the proportion of CHD IE ranging from 20.1–36.7% [14–16]. RHD once the most common underlying heart disease in the 1990s according to Chao et al.’ s report [17] was gradually decreasing in our study, which was consistent with other researches from China [6, 15, 16, 18]. These findings were some different from developed countries [6]. Researches from developed countries presented that DVD, PVE and implantable electronic devices related IE had gradually increased and replaced RHD as the leading heart disease [1, 4, 10]. This difference may contributed to the late diagnosis for CHD, the low screening rate for newborn and the low proportion of surgical treatment when they were young in China [6].

According to Song Bing’s report [19], there were 35.6% patients diagnosed as CHD after 18 years old, and among patients with septal defect, 60.1% were diagnosed in adulthood, and only 6.7% were diagnosed in infancy. Data from Guangdong congenital heart defects monitoring network showed that the cumulative incidence of CHD in Guangdong province increased from 3.74 to 11.29 per thousand per year from 2004 to 2016 and the number of adult CHD gradually increased, although it currently accounts for only 21%, but the growth is nearly 18% [20]. According to Lai Xiaojin’s report [20] based on a 2356 cases analysis, the rate of adults with CHD has an increasing trend. The same phenomenon was seen from developed countries. This might due to that the symptoms and signs were not obviously during adolescence for some non-severe CHD, and they failed to be identified due to the restrictions of local medical and economic conditions in our region.

IE was an important complication for patients with CHD [21, 22]. According to the published reports, the risk of developing into IE increased in patients with CHD [21, 23, 24]. Darren et al. [24] found that the IE risk exceeded 100 times in patients with ACHD compared to that of the general population and 2.5 times that of children with CHD [24, 25]. The proportion of patients with CHD in our study was as high as 25.8%.

TEE was performed in only 12.8% patients with IE in our study, as most patients diagnosed after TTE did not perform TEE routinely. Compared to the literature published from China on IE, the rate approached to other studies in our region [14–16]. But it was significantly lower than that reported by other studies from developed countries with 74–100% [26, 27]. However, according to the 2015 European Association of Cardiology guidelines on IE, for TTE positive patients, TEE should be performed to exclude perivalvular complications [1]. The low utilization rate of TEE could directly resulted in the low detection rate of perivalvular complications such as valvular perforation and perivalvular abscess. Besides, this could cause the missed diagnoses of IE in patients with unobvious valvular lesions or with basic valvular lesions. This underlined that we still need to improve the use of TEE in IE diagnoses to reduce missed diagnoses.

The positive rate of blood culture in our study was lower compared with other studies from developed countries, where the rate of positive blood culture varied from 83 to 96% [2, 10, 13, 28] .However, the rate was approximate to other studies from China varied from 38.5–70.1% [14, 15, 18]. The low microbiology detection rate could be related to the extensive use of antibiotics before blood culture, and the proportion of patients with prior antibiotics use was as high as 81.7% in our study. Besides, as a tertiary hospital, most patients in our hospital had been referred from other medical institutions. They usually had a history of antibiotic treatment previous to the blood cultures. What’s more, the blood cultures of the HACEK group, serology tests for *mycoplasma*, *bartonella, legionella* and the polymerase chain reaction (PCR) were not performed in our hospital. Therefore, we suggested that patients with suspected bloodstream infection should receive blood cultures routinely before the use of antibiotics.

In our study, *Streptococci* was the main pathogen accounting for 24.6%, followed by *Staphylococci* accounting for 19.7%, and these were the same as reports in the 1990s from China [17]. This phenomenon was similar to other developing countries [5, 6] but was different from developed countries [4]. For IE in developed countries, the proportion of *Staphylococci* increased gradually and became the main pathogenic bacteria [4, 6]. The increase in *Staphylococcus* IE was mainly due to the high incidence of intravenous drug addicts, hemodialysis patients and elderly patients with comorbidities [3, 4, 29, 30]. However, in our study, the proportion of patients with intravenous drug addicts, hemodialysis patients and octogenarians was lower compared with developed countries. Besides, most of patients in our study were community origin IE, and *Streptococcus* IE was the main for community origin IE according to the previous reports [16].

*Enterococci* IE was less common and the rate was stable in our study. This were consistent with another report from our region [18]. In this report, *Enterococci* was identified in 5 patients (2.9%) and the proportion was stable during 2008 to 2015 [18]. However, this was quite different from developed countries [6]. In developed countries, *Enterococci* IE was more common and was the third leading cause of IE after *Staphylococci* and *Streptococci* ranging between 7 and 18% [9, 10, 28]. According to the previous report, *Enterococci* IE was most frequently seen in elderly men with a relatively low short-term mortality [31].

According to the published studies, older age, prosthetic valve IE, heart failure, septic shock, *Staphylococcus aureus*, and large vegetation were predictors of poor outcome in patients with IE [1, 4]. In our study, we found that hemodialysis, pulmonary hypertension, Pitt score ≥ 4 and vegetation length>30 mm were independent risk factors for in-hospital mortality. The risk of in-hospital mortality in patients with hemodialysis increased by 4.697 times and in patients with pulmonary hypertension, the risk of in-hospital mortality increased by 5.308 times.

There are several limitations in our study. First, as a retrospective study, there exists information bias, and we are unable to obtain information on the patient exposure to dental procedures and the use of antibiotic prophylaxis. Second, the study used a single-center cohort in a tertiary teaching hospital with possible selection bias that could not represent the entire Chinese condition. Finally, as a nonrandomised study, there were associated limitations and selection bias affecting comparisons between the in-hospital outcomes. Therefore, we suggest multiple-center prospective cohort studies performed in our region.

## Conclusions

In conclusion, this study is currently a large sample research in IE from our region. It presents that the mean age of IE onset has being older, congenital heart disease is the predominant cardiac disease and *Streptococci* is still the predominant pathogen. Attention should be paid to the management of congenital heart disease especially among adult with congenital heart disease. Besides, it shows that patients with hemodialysis, pulmonary hypertension, Pitt score ≥ 4 and vegetation length>30 mm have an worse in-hospital outcome. Our findings will have an important impact in the improvement both in the diagnosis and treatment of IE in our region in the future.

## Data Availability

The datasets used and analyzed during the current study are available from the corresponding author on reasonable request.

## Abbreviations

IE: Infective endocarditis
PVE: Prosthetic valve endocarditis
RHD: Rheumatic heart disease
CHD: Congenital heart disease
ESC: European Society of Cardiology
TTE: Transthoracic echocardiography
TEE: Transesophageal echocardiography
SD: Standard deviation
IQR: Interquartile range
OR: Odds ratios
HR: Hazard ratio
CI: Confidence interval
